# COVID-19 clinical rebound after treatment with nirmatrelvir/ritonavir

**DOI:** 10.21203/rs.3.rs-4497916/v1

**Published:** 2024-06-21

**Authors:** Daniel Camp, Matthew Caputo, Fabiola Moreno Echevarria, Chad J. Achenbach

**Affiliations:** Northwestern University; Northwestern University; Northwestern University; Northwestern University

**Keywords:** nirmatrelvir/ritonavir, rebound, COVID-19, SARS-CoV-2, anti-viral

## Abstract

**Background:**

Nirmatrelvir/ritonavir (NM/r) is a safe and effective oral antiviral therapeutic used for treatment of mild-to-moderate COVID-19. Case reports described a clinical rebound syndrome whereby individuals experience a relapse of symptoms shortly after completing successful treatment. There is a lack of information on frequency of COVID-19 rebound after NM/r in routine clinical care, contributing factors, and clinical outcomes.

**Methods:**

We reviewed electronic medical records to verify COVID-19 diagnosis, symptoms, and treatment with NM/r from January-June 2022. We defined COVID-19 clinical rebound as clear improvement in symptoms followed by recurrence or worsening of symptoms within 30 days of a five-day course of NM/r.

**Results:**

We studied 268 adults with median age 57 (IQR 47, 68), 80% White race, 85% non-Hispanic ethnicity, 55% female, 80% vaccinated and boosted against SARS-CoV-2, and 68% with any co-morbidity. Sixteen (6.0%) of studied patients were determined to have COVID-19 clinical rebound. The median time from starting NM/r to rebound was 11 days (IQR 9, 13). Notable demographic and clinical factors with higher proportion (not statistically significant) among COVID-19 rebound patients were female sex (75% rebound vs 54.5% no rebound), Black race (12.5% rebound vs 4.9% no rebound), presence of at least one co-morbidity (81.3% rebound vs 67.5% no rebound), and lack of prior SARS-CoV-2 infection (100% rebound vs 92.9% no rebound). Only one patient (6.25%) was hospitalized after COVID-19 rebound.

**Conclusions:**

COVID-19 clinical rebound after treatment with NM/r is mild with favorable outcomes and more common than previously reported from real-world clinical care studies.

## Background

Nirmatrelvir/ritonavir (NM/r) (Paxlovid) is an oral antiviral therapy that has been granted FDA approval for the treatment of mild-to-moderate COVID-19 with large cohort studies and randomized controlled trials demonstrating reductions in hospitalization or death^1–3^ or subsequent complications^4^ and lowering of SARS-CoV-2 viral load^5^ among unvaccinated individuals with non-Omicron variants of SARS-CoV-2. After FDA Emergency Use Authorization (EUA) approval, NM/r was widely used in outpatient clinical practice and many providers observed symptomatic COVID-19 and SARS-CoV-2 antigen testing rebound several days after completing a five-day course of NM/r^6,7^. Early case reports described individuals experiencing a relapse or recrudescence of COVID-19 symptoms or new symptoms after an initial clinical improvement on NM/r^8,9^. One observational EMR-based study of outpatients with mild COVID-19 found the 30-day cumulative incidence of symptom rebound after NM/r treatment was only 0.8%^10^. In one carefully conducted cohort study with frequent symptom assessments, the 10-day cumulative incidence of COVID-19 symptom rebound (32%) was significantly greater in the treated group than the incidence (20%) among controls^11^, while in other studies the 28-day cumulative incidence of COVID-19 symptom rebound was 19 to 25% regardless of anti-SARS-CoV-2 therapy^12,13^. Thus, there remains uncertainty of the incidence of COVID-19 symptom rebound that is brought to medical attention and requires outpatient clinical assessment after NM/r treatment in the real-world.

There have been no clear clinical factors associated with COVID-19 symptom rebound and the current predominant, yet unproven, hypothesis is that COVID-19 rebound may occur due to a weaker or delayed immune response as NM/r suppresses SARS-CoV-2 early in infection; however, a thorough immunology study of patients with COVID-19 rebound observed robust antibody and T-cell immune responses regardless of NM/r treatment^14^. Furthermore, prior studies did not properly assess whether differing levels of immunity from vaccine or prior SARS-CoV-2 infection were associated with COVID-19 rebound after NM/r. In terms of outcomes, COVID-19 rebound disease has been mild^7,10,15^ and less than 1% have been hospitalized during rebound, but prior studies have not fully assessed healthcare system visits including telemedicine, emergency departments, urgent care centers, and ambulatory clinics^10,16^.

We performed this study from a large healthcare system in Chicago to provide additional real-world research on frequency and timing of COVID-19 clinical rebound, potential contributing factors, and outcomes among patients with varying levels of immunity due to vaccine or prior SARS-CoV-2 infection.

## Methods

We conducted a clinical cohort study utilizing the Northwestern Medicine Enterprise Data Warehouse (NMEDW), an integrated repository of clinical data sources across the Northwestern Medicine (NM) system^17^. First, we identified adults (18 years or older) who had been prescribed NM/r by a NM provider between January 1, 2022 and May 31, 2022. We then reviewed electronic medical records (EMR) and included patients for whom we were able to verify COVID-19 diagnosis, determine symptoms, and confirm treatment with NM/r. We excluded individuals who did not have confirmed COVID-19 infection by testing (either antigen or PCR) or did not complete a full five-day course of NM/r. Finally, we evaluated for COVID-19 clinical rebound by reviewing provider and support staff EMR documentation of follow-up telehealth or in-person clinical encounters. COVID-19 clinical rebound was defined as EMR documentation of provider-confirmed clinical improvement in COVID-19 symptoms followed by recurrence or worsening of prior COVID-19 symptoms or new symptoms consistent with COVID-19 after successful completion of NM/r and within 30 days of starting therapy.

Vaccine status was determined through NMEDW. Patients with at least one SARS-CoV-2 vaccine dose following a two-dose mRNA series (Pfizer or Moderna) or one-dose Janssen series were categorized as “boosted”, those with only a two-dose mRNA series or one-dose Janssen series were categorized as “fully vaccinated”, and all others were categorized as “not fully vaccinated”. Comorbidities were determined from ICD9/ICD10 diagnosis codes assigned at any time prior to starting NM//r. Obesity was determined by either coding or vitals indicating BMI > 30 within two years prior. Prior SARS-CoV-2 infection was determined by having any positive PCR tests at least 90 days prior to starting NM/r, with NMEDW capturing data as far back as March 2020. Health system encounter visits within 60 days of NM/r initiation were pulled from NMEDW, with chart review to verify whether the encounters listed COVID-19 as the primary diagnosis.

Descriptive statistics were utilized to describe patient characteristics, symptomology, and relapse clinical outcomes. This included proportions for categorical data and medians with interquartile ranges (IQRs) for continuous data. Bivariate comparative analyses between those with and without COVID-19 rebound were conducted using Fisher’s exact tests. Multivariable analyses were not performed due to the small number of COVID-19 rebound events. Analyses were performed in R (R version 4.2.1, https://www.r-project.org) and SAS (SAS Institute, Inc., Cary, NC) version 9.4.

## Results

We included 268 adults who completed a five-day course of NM/r for outpatient treatment of mild COVID-19. The median age of patients was 57 years (IQR: 45, 69 years). We determined that 16 (6.0%) patients had COVID-19 clinical rebound within 30 days after completion of NM/r (Table 1). In the rebound group, 12 (75%) patients were female, compared to 134 (53.2%) in the no-rebound group (p = 0.12). Two (12.5%) patients identified as Black, compared to 11 (4.4%) in the no-rebound group (p = 0.18). None of the patients in the rebound group had previously tested positive for SARS-CoV-2 at NM, while 18 (7.1%) patients in the no-rebound group had prior positive testing (p = 0.61). Thirteen (81.3%) rebound patients had at least one comorbidity, compared to 168 (66.7%) in the no-rebound group (p = 0.29). All 16 (100%) of the COVID-19 rebound patients had been fully vaccinated and 12 (75%) had also received at least one booster vaccine dose, compared to 236 (93.6%) fully vaccinated and 203 (80.6%) vaccinated and boosted in the no-rebound group. None of the univariable statistical comparisons of demographic and clinical factors in Table 1 reached statistical significance (p-value < 0.05).

**Figure 1:**
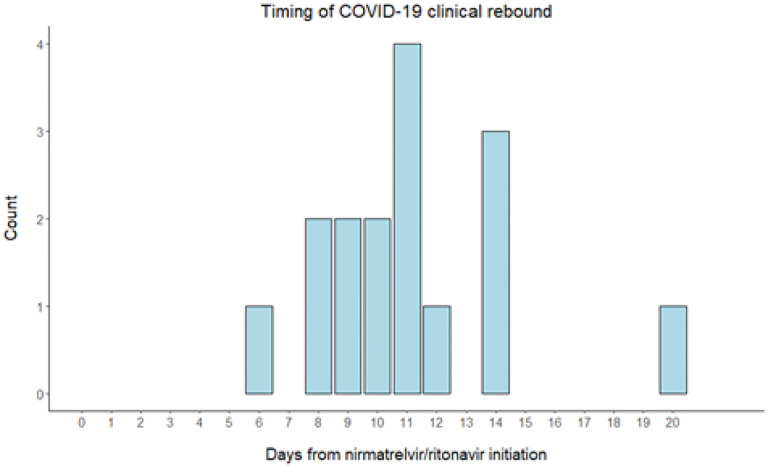
Time from initiation of nirmatrelvir/ritonavir to COVID-19 clinical rebound

The median time from initiation of nirmatrelvir/ritonavir to COVID-19 clinical rebound was 11 days (IQR: 9,13). A graph of timing of COVID-19 clinical rebound can be found in Figure 1.

The most common symptoms experienced during COVID-19 rebound were nasal congestion (68.8%) and cough (62.5%) (Table 2). COVID-19 rebound was brought to clinical attention and assessed via telemedicine (37.5%), immediate care (25%), and ambulatory (25%) visits (Table 2). Two COVID-19 rebound cases led to moderate-severe disease resulting in an emergency department visit with hospital admission, and a telemedicine assessment with subsequent immediate care center visit. A case-by-case description of demographics, clinical characteristics, and healthcare system visit for each of the 16 cases of COVID-19 rebound can be found in Table 3.

## Discussion

We found that 6% of patients included in our study who completed a five-day course of NM/r developed COVID-19 clinical rebound leading to healthcare provider assessment within 30 days of treatment. Rebound symptoms were predominantly cough and nasal congestion occurring from 1 to 15 days following completion of NM/r. The timing of rebound was similar to prior case reports and cohort studies^18,19^; however, our cumulative incidence was higher than 0.8% reported in one prior clinical care study^10^ of outpatients with mild COVID-19. A rigorously conducted prospective study found a considerably higher cumulative incidence of symptom rebound after NM/r of 18.9%, and incidence in those not treated in this study was similar to our study at 7%^12^. Several studies have observed frequency of symptom rebound similar between those who did and did not receive NM/r^2,20^. One study of the placebo arm of ACTIV clinical trials found higher COVID-19 symptom rebound at 26%^13^. These results suggest symptom recrudescence or worsening is common in the natural course of resolving COVID-19 disease regardless of anti-viral treatment. These discrepancies are likely due to study methodology whereby carefully conducted cohort studies performed daily or every other day symptom and nasal swab virology assessments. Thus, they were more likely to pick up mild and/or transient symptom recurrence and overestimate the rate of COVID-9 rebound. Our study relied on both patient and provider reporting to the NM healthcare system and recording in medical records which may not have occurred for many patients with mild rebound illness or who sought care outside of our clinics. In summary, we estimate that COVID-19 symptom rebound frequency after NM/r lies between 6 and 26%, depending on patient characteristics, severity of symptoms, and ease of reporting or symptom assessment.

In terms of risk factors, like other studies, we did not find any significant associations between demographic or clinical characteristics and COVID-19 symptom rebound after NM/r. An interesting observation was an indication that patients with lower immunity to SARS-CoV-2 (less vaccine boosting, lack of prior infection, and co-morbidities) experienced higher frequency of COVID-19 symptom rebound after NM/r. This supports the current hypothesis that NM/r anti-viral activity and reduction of SARS-CoV-2 in tissues is enhanced by strong host immunity. Thus, COVID-19 viral and/or symptom rebound is more likely to occur among those who fail to adequately clear SARS-CoV-2 virus in the upper respiratory tract in a dynamic process. Anti-viral therapies, such as NM/r, inhibit viruses and reduce the potential for severe complications such as pneumonia; however, viral clearance takes time and virus replication after completion of NM/r could increase due to several potential mechanisms. This is supported by in a prior study with frequent early PCR and viral culture sampling that found significantly greater virologic rebound and prolonged shedding of replication-competent SARS-CoV-2 virus after NM/r treatment compared to no COVID-19 therapy^21^. Contrary to this hypothesis was a carefully conducted immunology study that found individuals treated with NM/r for COVID-19 had similar robust humoral and T-cell immune responses^14^ regardless of whether they experienced rebound; however, it is unclear whether viral dynamics and clearance were different within upper respiratory tract tissues. In addition, while resistance gene mutations encoding in SARS-CoV-2 protease have been suggested as a cause of viral rebound, this has not yet been observed^14,18^

Other potential risk factors we identified for clinical rebound after NM/r were female sex, Black race, having comorbidities, and systemic symptoms. Similar to other studies^11^, a non-significant, but higher proportion of patients with rebound had systemic symptoms of fever, shortness of breath, myalgia, and headache with their presenting COVID-19 illness, possibly indicating a more severe presentation. These findings are also likely to be the result of higher health awareness and health-seeking behaviors for certain demographic groups. Black race and certain comorbidities have been associated with more severe COVID-19 infection throughout the pandemic^22,23^. It is unlikely that there is a biological basis to an association with Black race and a higher risk of rebound – rather, it is likely due to the higher prevalence of certain comorbidities among Black individuals^24^. This is potentially due to multiple factors, including socioeconomic status and access to and treatment within the healthcare system. Interestingly, this lack of access to the healthcare system would be expected to underestimate any potential association between Black race and rebound; this potential association should be investigated further in larger studies. As briefly mentioned above, comorbidities could contribute to rebound for several reasons -- a weaker immune response to SARS-CoV-2 infection and lower viral clearance/control, greater susceptibility to respiratory infection or multi-organ involvement, and greater engagement with the healthcare system.

As reported in many prior studies and case reports, we also found that rebound COVID-19 is generally mild, managed on an outpatient basis, and does not lead to poor health outcomes. Only one case of NM/r rebound in this study led to an ED visit and hospital admission, with the rest managed by ambulatory care or telemedicine. Thus, when weighing benefits and risks of NM/r treatment in light of the potential for rebound COVID-19, recent real-world research continues to show clear benefits of NM/r treatment in high-risk populations regardless of immunity from vaccine or natural infection^2^.

## Limitations

This study was subject to several limitations since it was observational, EMR-based, and relied on adequate documentation from providers and healthcare staff to meet our definition of COVID-19 rebound. Thus, there was the possibility of misclassification and underreporting of rebound symptoms and certain important risk factors such as SARS-CoV-2 vaccination or previous COVID-19. In addition, we were unable to assess patient adherence and had to assume that patients who started the regimen completed the entire 5-day course. However, our study of a diverse subset of patients in a large metropolitan healthcare system found that NM/r rebound is occurring, tends to be mild, and identified potential risk factors and rebound mechanisms to be further investigated.

## Conclusion

COVID-19 clinical rebound after treatment with NM/r is more common than previously reported in the peerreviewed published literature. Rebound is generally mild and has favorable outcomes. Potential risk factors for experiencing rebound are female sex, Black race, systemic symptoms with COVID-19 illness, the presence of comorbidities, and lower SARS-CoV-2 immunity due to under vaccination or lack of prior COVID-19. These factors should be investigated further in larger observational studies.

## Figures and Tables

**Table 1:** Characteristics (N=268) of adults who did (n=16) and did not (n=252) experience COVID-19 clinical rebound within 30 days after completing NM/r.

Variable			
	Total	Rebound (n=16)	No Rebound (n=252)
**Age**	57 (45, 69)	52 (44.5, 65.5)	57.5 (47, 68)
**Sex**			
Female	146 (54.5)	12 (75.0)	134 (53.2)
Male	122 (45.5)	4 (25.0)	118 (46.8)
**Race**			
White	215 (80.2)	11 (68.8)	204 (81.0)
Black or African American	13 (4.9)	2 (12.5)	11 (4.4)
Asian	11 (4.1)	1 (6.3)	10 (4.0)
American Indian or Alaskan Native	1 (0.4)	0 (0)	1 (0.4)
Other	16 (6.0)	0 (0)	16 (6.4)
Unknown	12 (4.5)	2 (12.5)	10 (4.0)
**Ethnicity**			
Hispanic or Latino	15 (5.6)	0 (0)	15 (6.0)
Not Hispanic or Latino	227 (84.7)	16 (100.0)	211 (83.7)
Unknown	26 (9.7)	0 (0)	26 (10.3)
**Previous SARS-CoV-2 Infection**	18 (6.7)	0 (0)	18 (7.1)
**Diagnosis Method**			
Molecular	146 (56.4)	10 (62.5)	136 (56.0)
Home Antigen	113 (43.6)	6 (37.5)	107 (44.0)
**Symptoms at NM/r initiation**	257 (97.4)		
Cough	177 (66.0)	10 (62.5)	167 (66.3)
Nasal Congestion/Runny Nose	135 (50.4)	9 (56.3)	126 (50.0)
Fever/Chills	116 (43.3)	8 (50)	108 (42.9)
Sore Throat	112 (41.8)	6 (37.5)	106 (42.1)
Myalgia	83 (31.0)	6 (37.5)	77 (30.6)
Headache	72 (26.9)	7 (43.8)	65 (25.8)
Fatigue	71 (26.5)	3 (18.8)	68 (27.0)
Shortness of Breath	23 (8.6)	3 (18.8)	20 (7.9)
Nausea/Vomiting/Diarrhea	22 (8.2)	2 (12.5)	20 (7.9)
Abnormal Taste or Smell	9 (3.4)	1 (6.3)	8 (3.2)
Ear Pain	3 (1.1)	0 (0)	3 (1.2)
Chest Pain	2 (0.8)	0 (0)	2 (0.8)
**Vaccination Status**			
Fully Vaccinated and Boosted	215 (80.2)	12 (75.0)	203 (80.6)
Fully Vaccinated, No Boost	37 (13.8)	4 (25.0)	33 (13.1)
Not Vaccinated	16 (6.0)	0 (0)	16 (6.4)
**Time From Symptoms to** NM/r **initiation (days)**	1 (1,3)	1 (1,3)	1 (1,3)
**Time From Positive Test to NM/r Initiation (days)**	1 (0,1)	1 (0,1)	1 (0,1)
**Time From Last Vaccine Dose to Positive Test (days)**	167 (140, 201)	164.5 (145.5, 180.5)	168 (139, 204)
**Comorbidities**			
Obesity	117 (43.7)	7 (43.8)	110 (43.7)
Diabetes	34 (12.7)	2 (12.5)	32 (12.7)
HIV	12 (4.5)	2 (12.5)	10 (4.0)
Chronic Liver Disease	11 (4.1)	2 (12.5)	9 (3.6)
Renal	18 (6.7)	0 (0)	18 (7.1)
SCT	2 (0.8)	0 (0)	2 (0.8)
Hematologic Malignancy	8 (3.0)	0 (0)	8 (3.2)
Asthma	35 (13.1)	4 (25.0)	31 (12.3)
COPD	1 (0.4)	0 (0)	1 (0.4)
Cancer	44 (16.4)	3 (18.8)	41 (16.3)
Cardiovascular Disease	58 (21.6)	6 (37.5)	52 (20.6)
Hypertension	100 (37.3)	5 (31.3)	95 (37.7)
Immunodeficiency	34 (12.7)	3 (18.8)	31 (12.3)
Comorbidities (Any)			
Yes	181 (67.5)	13 (81.3)	168 (66.7)
No	87 (32.5)	3 (18.8)	84 (33.3)

**Table 2:** Clinical characteristics of patients who experienced COVID-19 rebound (N=16)

Variable	N (%) or Median (IQR)
**Days from NM/r therapy initiation to COVID-19 rebound**	11 (9,13)
**Symptoms (during COVID-19 rebound)**	
Nasal Congestion/Runny Nose	11 (68.8)
Cough	10 (62.5)
Sore Throat	4 (25.0)
Headache	4 (25.0)
Fever/Chills	3 (18.8)
Body Aches	3 (18.8)
Fatigue	3 (18.8)
Nausea/Vomiting/Diarrhea	2 (12.5)
Shortness of Breath	2 (12.5)
Abnormal Taste or Smell	1 (6.3)
**Healthcare System Encounters (during COVID-19 rebound)**	
Hospital Admission	1 (6.3)
Immediate Care Center	4 (25)
Ambulatory/Outpatient	4 (25)
Emergency Department	1 (6.3)
Telemedicine	6 (37.5)

**Table 3:** Summary Table of Rebound Cases

Case	Age	Sex	Race	Vaccination status	Comorbidities	Days from NM/r initiation to rebound	Rebound symptoms	Healthcare encounter
1	44	F	Asian	Boosted	Obesity, DM, CLD	20	Sore throat, nasal congestion, headache	telemedicine
2	87	M	White	Boosted	DM, Asthma, Cancer, CVD, HTN, Immune Disorder	11	Cough. nasal congestion, body aches, headache	telemedicine
3	42	F	White	Boosted	Cancer	11	Nasal congestion, body aches	telemedicine
4	45	F	Unknown	Boosted	Obesity	14	Cough	ICC
5	53	M	White	Fully Vaccinated	HTN	6	Cough, nasal congestion, headache	ICC
6	46	F	White	Boosted	HIV, Asthma, CVD	10	Nasal congestion, nausea	ED
7	53	F	White	Boosted	Obesity, Asthma, CVD, HTN	12	Cough, fever, body aches	telemedicine
8	72	F	White	Boosted	CLD, HTN	8	Cough, nasal congestion, headache	ambulatory
9	66	M	White	Boosted	CVD	8	Cough, nasal congestion, fever	telemedicine
10	50	F	Unknown	Boosted	HIV, HTN	14	Sore throat, nasal congestion, nausea, dyspnea, fatigue	ambulatory
11	51	F	Black or African American	Boosted	Obesity, CVD	9	Dyspnea, fatigue	hospitalized
12	62	F	Black or African American	Fully Vaccinated	Obesity	9	Cough, fever, fatigue	ICC
13	68	F	White	Boosted	-	14	Cough, nasal congestion	telemedicine
14	65	F	White	Boosted	Immune Disorder	10	Sore throat, cough	ambulatory
15	30	M	White	Fully Vaccinated	Obesity, Cancer	11	Cough, nasal congestion	ICC
16	39	F	White	Fully Vaccinated	Obesity, Asthma, CVD, Immune Disorder	11	Sore throat, nasal congestion, anosmia	ambulatory

## Data Availability

Data were collected from the *Northwestern Medicine Enterprise Data Warehouse. De-identied data can be made available upon reasonable request*.
